# Chronic Diarrhæa

**Published:** 1847-01

**Authors:** 


*Chronic Diarrhœa.*—A. B., aged one year and a half, had remittent fever in February—recovered in six weeks; since which time the bowels have generally been loose, and the child has emaciated rapidly, and having very little appetite, lives almost exclusively upon bread and molasses. Stools are now frequent (12 in a day,) and very offensive. MM. ℞. Pulv. Doveris gr. vj. Pulv. Hyd. c. Creta gr. xxiv. m. ft. Pulv. No. xij. Cap. unum ter in die. Let the child eat soda biscuit and drink rice water, the later slightly alkalized by Carb Potass.

*Remarks by Prof. Gilman.*—This is one of the forms of that intestinal irritation of which, at this season, our present city presents so many. In treating them, the older practitioners relied upon alteratives and evacuants, and dreaded nothing so much as a sudden check to the discharges, which, as they supposed, was very likely to produce a determination to the brain and even fatal effusion. To support this view, cases were quoted where children in the very last stage of exhaustion from cholera infantum, died with (so called) hydrocephalus. Now I believe that this is all wrong—the pathology erroneous—the treatment mischievous.

First as to the pathology: do the hydrocephaloid symptoms result from congestion of the brain? I believe they do not, but are to be referred to that reaction after exhaustion, a knowledge of which we owe to Marshall Hall. In proof of this I could cite many cases where the symptoms gradually gave way under the use of brandy and arrow root, minute doses of opium, etc. As to the practice, I have checked the diarrhœa of infants hundreds of times, and never have I known this congestion of the brain to follow the suppression. So far am I from fearing this, that it is the indication I keep in view, the object I wish to accomplish; for this purpose I give Dover's powder with the mercurial, or if the stools are very frequent and profuse, I check them by laudanum and starch injections, and then give the alterative, which, thus given, will correct morbid secretion much better than when it is hurried through the bowels by a cathartic, or allowed to race its way through under the influence of excited peristalic action. Here then you have a general principal upon which to treat these irritations of the alimentary canal in children. Allay irritation and moderate peristalic action by one or more opiates, and then give your alterative, or combine the two together, which, as the opiate acts almost immediately and the alterative only after several hours, comes to the same thing.

*Scrofulous Swellings, Ophthalmia, and Cutaneous Eruptions.*—B. K., aged two and a half years, resides in Jersey City, healthy till last fall: then had (so called) tinea capitis, which her physician proposed to cure by a pitch plaster; it was not applied. Scrofulous tumors next appeared, one of which, behind the left ear, suppurated, discharge free and very fœtid; (scrofulous) ophthalmia attacked her soon after and she had an eczema of the face and behind the ear. *Present condition.* Ophthalmia, evidently scrofulous, is on the decline, so is the eruption of the scalp, which is impetigo. Behind the left ear there is a sinus, the two openings about an inch apart; bowels rather torpid, stools very offensive, appetite irregular. MM. Lay open the sinus and dress it with lint—regulate the diet and give rhubarb 8 grs., soda 6 grs., every morning. Bath (nearly cold) daily, and be rubbed with the salt towel.

*Remarks by Prof. Gilman.*—All this child's ailments are dependent on a scrofulous taint of constitution, and the remedies, to have any thing more than a temporary success, must be directed to this state of the general system and not to the particular manifestations which may chance to give most trouble at the moment. Plasters to the head, salves behind the ears, and washes for the eyes are all futile; correct the secretions, invigorate the general health, and when this is done, neither plasters, salves, no wash will be required, the disease (scrofula) will be cured, and the symptoms (eczema, ophthalmia, and glandular swellings,) will disappear.

*Dysmenorrhœa with Anœmia.*—B. B., aged 17. Has enjoyed excellent health till within a few months, during which she has suffered much from dysmenorrhœa, headache and extremely violent pain in the right hypochondrium. *Present state.* Countenance pallid, eye heavy, mouth dry, tongue slightly furred, pulse quick and harsh, action of the heart violent, and as she says, much augmented by going up stairs. Bruit and soufflet very evident at the base of the heart, appetite lost, bowels very costive. MM, an alœtic purgative every night, so as to secure a free evacuation of the bowels daily, diet rather light, return in a week. *Remarks.* This case presents some of the features by which practitioners are often led to deplete anæmic patients, and of course destroy them. Had this patient a hot skin, and great tenderness on pressure in the hypochondriac region where pain is now felt, symptoms which we often see in such a case as hers, a phlegmasia might be diagnosticated, and the mistake once made and acted on, the complete though temporary relief that would follow a free bleeding, would be sure to confirm the physician in his error; thus bleeding would follow bleeding till the patient was litterally bled to death. *I have seen this*; I have seen the post mortem examination of a patient, who was drained of almost every drop of blood in her body, for a supposed phlegmasia. *There was no trace of inflammatory disease in any part.* In all these anæmic cases iron is the great remedy; but to prepare the way for it, to fit the system for its use, constipation must be obviated and the secretions corrected, then *iron will cure.* This patient will probably be obliged to take aloes for a week or two before she will bear iron.

